# The Effect of High-Frequency Vibration on Tooth Movement and Alveolar Bone in Non-Growing Skeletal Class II High Angle Orthodontic Patients: Case Series

**DOI:** 10.3390/dj8040110

**Published:** 2020-10-01

**Authors:** Tarek El-Bialy

**Affiliations:** Division of Orthodontics, Katz Group Centre for Pharmacy and Health Research, School of Dentistry, University of Alberta, Edmonton, AB T6G 1C9, Canada; telbialy@ualberta.ca; Tel.: +1-780-492-2751

**Keywords:** high frequency vibration, clear aligners, skeletal Class II, non-surgical treatment, malocclusion

## Abstract

This study presents a novel technique utilizing high-frequency vibration to shorten treatment time and preserve alveolar bone in challenging orthodontic cases that have been treated with Invisalign^®^ clear aligners. Four non-growing orthodontic patients (age range 14–47 years old) with Class II skeletal patterns (convex profiles with retrognathic mandibles) who sought correction of their crowded teeth and non-surgical correction of their convex profiles were included in this study. These patients were treated using Invisalign clear aligners together with high-frequency vibration (HFV) devices (120 Hz) (VPro5™) that were used by all patients for five minutes per day during active orthodontic treatment. Vertical control and forward rotation of the mandible for each patient was achieved through pre-programming the Invisalign to produce posterior teeth intrusion. Successful forward rotation of the mandibles achieved in all patients led to improvement of their facial convex profiles (apical base relationship (ANB) improved 2.1 ± 0.5 degrees; FMA (Frankfurt mandibular plane angle) improved 1.2 + 1.1 degrees). Dental decompensation was achieved by lingual tipping of the lower incisors and palatal root torque of upper incisors. The use of HFV together with Invisalign facilitated achieving these results within a 12 ± 6 months period. In addition, more bone labial to the lower incisors after their lingual movement was noted. In conclusion, the use of HFV concurrent with SmartTrack Invisalign aligners allowed complex tooth movement and forward mandibular projection without surgery in non-growing patients with skeletal Class II relationships. The clinical impact and implications of this case series are: (1) the use of HFV facilitates complex orthodontic tooth movement including posterior teeth intrusion and incisor decompensation; (2) forward mandibular projection of the mandible and increased bone formation labial to lower incisors can be achieved in non-growing patients that may minimize the need for surgical intervention in similar cases or gum recession due to lower incisors labial inclination.

## 1. Introduction

Orthodontic treatment using clear aligners has become the most sought-after treatment option for both adults and teenagers. Clear aligners are chosen mainly for esthetics, better oral hygiene, and no food limitations or constriction like with fixed orthodontic appliances [1,2]. Traditionally, it was recommended that patients wear aligner sets for fourteen days before they changed to the next set in the series [3]. However, in an attempt to accelerate tooth movement of clear aligners, and with the development of advanced materials (such as Align technology’s SmartTrack^®^ material) used in fabricating clear aligners by many companies, many patients were able to change their aligners every seven days [1]. The reduced number of days per set of aligners was contingent upon the patient wearing the aligners for minimum of 22 h per day [1]. This presents another challenge since many patients are not compliant to the level of wearing their aligners 22 h per day [4,5]. For this reason, the proposed seven-day aligner change by Align technology was not always easy to implement in daily practice, particularly with regard to the cases of increased complexity that are now being treated by Invisalign SmartTrack^®^ clear aligners [6,7,8]. Combining these two factors (compliance in amount of time wearing aligners and complexity of cases currently being treated), the treatment time involved to treat complex cases has become unpredictable and unnecessarily long. With this in mind, many techniques have been proposed to accelerate tooth movement especially in complex or challenging cases [6,7,8,9,10,11,12,13]. These techniques include, but are not limited to, micro-osteoperforation, photobiomodulation, low- and high-frequency vibrations, laser, and low intensity pulsed ultrasound [6,7,8,9,10,11,12,13,14]. The effectiveness of low vibration has been previously questioned and recent literature showed its ineffectiveness [14]. Comparison of both low- and high-frequency vibration showed that HFV is significantly more effective in accelerating tooth movement compared to low frequency vibration [15].

The common biological effect of these techniques is the activation of local cytokines that accelerate osteoclastic activities that in general activate osteoblasts as well [6,7,8,9,10,11,12,13]. However, in most of these reported techniques, there are some limitations including long daily application times. Longer daily treatment times result in weaker patient compliance and, consequently, the effectiveness of the acceleration technique is diminished [6,7,8,9,10,11,12,13]. High-frequency vibration (HFV) has shown to enhance tooth movement when applied only for 5 minutes per day. It has been shown to enhance both osteoclastic and osteoblastic activities as well as increase bone density in both animal and human patients [15,16,17,18,19,20,21,22,23,24]. It has been suggested that the effectiveness of HFV in accelerating tooth movement is due to its enhancement of bone remodeling [15,16,17,18,19,20,21,22,23,24]. A recent study showed that HFV has an additive effect on PGE2 and RANKL on human periodontal ligament cells [21]. The inducing effect of RANKL expression in human PDL cells is suggested to be mediated by activating the cyclooxygenase pathway [22]. Moreover, a recent study reported that high-frequency vibration synergistically enhances osteoclastogenesis and osteoclast activity via NF-κB activation, which leads to alveolar bone resorption and hence accelerated tooth movement, especially when a static force was continuously applied to the teeth [20]. In addition, a recent study shows that HFV induced differentiation of human periodontal ligament cells by increasing Col-I, Runx2, and Osterix growth factors [22]. Previous reports showed simple-to-moderate orthodontic cases treated by HFV or other accelerating techniques [16,17]. The aim of this study was to report on the effect of using HFV and Invisalign aligners on patients’ profiles with hyperdivergent mandibles in non-growing skeletal Class II cases. 

## 2. Materials and Methods

In this study, four non-growing patients with hyperdivergent and convex profiles due to reterognathic mandibles were treated with Invisalign clear aligners (Align technology, San Jose, CA, USA) and HFV was utilized using the VPro5 device (Propel Orthodontics, Milpitas, CA, USA) that delivers a frequency of 120 Hz and was prescribed for patients to use once a day for 5 minutes. All patients utilized vertical pull chin cup (Jaw Bra, Chanhassen, MN, USA) and they were instructed to change aligners whenever new aligners could fit without gaps between the teeth and aligners, and if no intolerable force/pressure was felt by use of the new aligners.

*Case Presentations*

These four cases had the common chief complaint of convex profiles with recessive chin. None of them were interested in any surgical option, while they were interested in improvement of their bites and profiles. The study was conducted in accordance with the Declaration of Helsinki, and the protocol was approved by the Ethics Committee of the University of Alberta (project identification code: Pro00074117, date of approval: 13 December 2017).

### 2.1. Case 1

**Figure 1:**
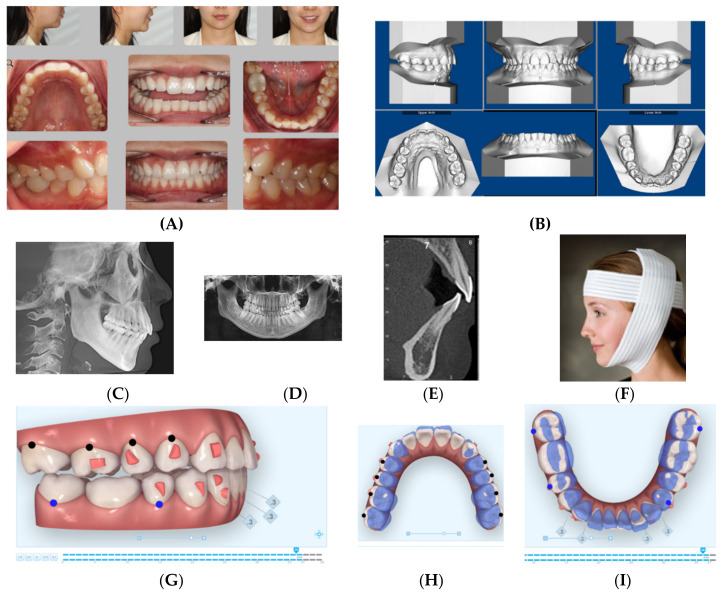

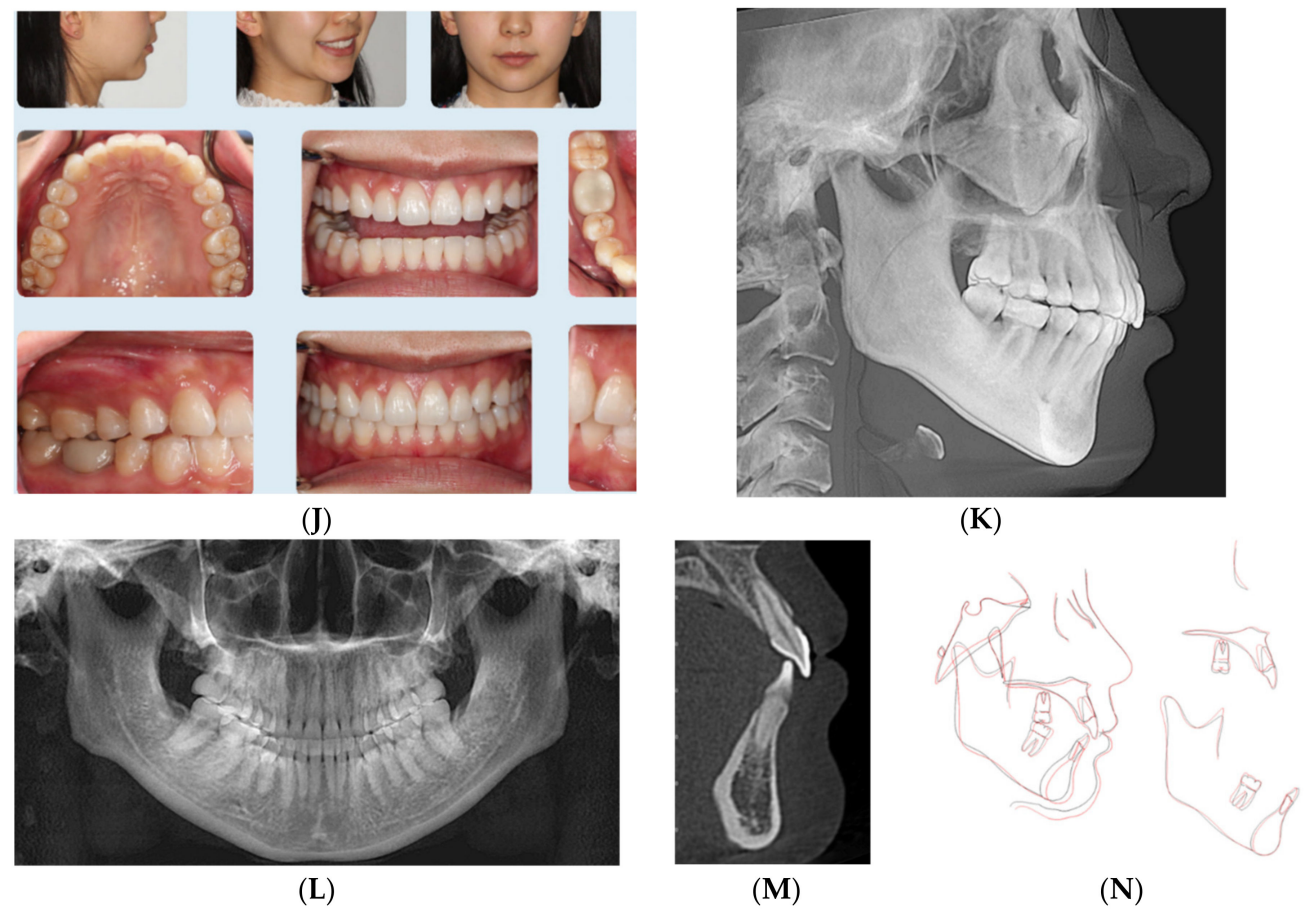
(**A**) Initial photographs showing the patient’s convex profile with recessive chin and anterior crowding. (**B**) Initial digital models showing left side Class II buccal occlusion with increased overjet 5 mm. (**C**) CBCT-driven cephalometric radiograph showing convex profile and recessive chin. (**D**) CBCT panoramic radiograph showing the patient is missing all third molars; no other noticeable intraoral bony lesion could be detected. (**E**) CBCT = driven sagittal screen of lower incisor showing its severe proclination with minimum bone appearing on most of the labial surface of the root. (**F**) A photo of the Jaw Bra (Chanhassen, MN USA). (**G**) Digital treatment plan (ClinCheck) showing posterior teeth distalization, mesio-bucaal rotation and intrusion to help forward rotation of the mandible to full Class I. (**H**) Upper arch digital treatment plan (ClinCheck) showing expansion (blue—initial teeth position, white—final teeth position). (**I**) Lower arch digital treatment plan (ClinCheck) showing expansion (blue—initial teeth position, white—final teeth position) to allow lower incisor intrusion and leveling /alignment of lower incisor crowding. (**J**) Final photos showing improved patient’s profile and chin projection as well as aligned upper and lower incisors and Class I buccal occlusion both sides. (**K**) CBCT-driven final cephalometric radiograph showing improved profile and chin projection (**L**). Final CBCT-driven panoramic radiograph. (**M**) Final CBCT-driven sagittal screen radiograph of upper and lower incisors showing improved bone labial to both upper and lower incisors. (**N**) Superimposition of cephalometric tracing of before (black) and after (red) treatment showing improved forward chin projection, palatal root torque of upper incisors, and profile and chin projection and lingual tipping of lower incisors.

This 28-year-old female presented with the chief complaint that she wanted to correct her crowded teeth (4 mm of crowding in the upper and lower arches) as well as her increased overjet, in addition to improving her convex profile, specifically her recessive chin. Her medical history was noncontributory, clinical records (Figure 1A) showed a convex profile with recessive chin. Intraoral photographs and digital models (Figure 1B) revealed a Class II division 1 subdivision left side (end-to-end left side buccal occlusion) and a right-side Class I. There was increased overjet (5 mm) and average overbite (30%), and her lower midline was deviated 2 mm to the left side. Cephalometric x-ray and analysis (Figure 1C and Table 1) revealed a skeletal Class II apical base relationship (ANB =7.9°) with compensated upper and lower incisors (U1-NA = 90.4° and L1 MP = 90°) (Figure 1C,E), recessive chin, and high mandibular plane angle (FMA = 38.1°). The patient was interested in improving her convex profile without surgical options. She had all third molars removed and no pathology was noted in her cone-beam computed tomography (CBCT)-driven panoramic radiograph (Figure 1D). The patient was provided with Invisalign clear aligners and HFV (VPro5) to use for 5 minutes every day. Additionally, the patient was provided with vertical-pull chin cup (Jaw Bra, Chanhassen, MN, USA) (Figure 1F) to use every night. The patient reported that she was able to change her aligners every 3–5 days without feeling too much pressure upon using new aligners.

After 6 months, the patient finished her active orthodontic treatment, and she was satisfied with overall results. Figure 1J–M show final clinical photos, the lateral CBCT-driven cephalometric radiograph and panoramic radiograph as well as the sagittal screen of upper and lower incisors. Figure 1N shows superimposition of before-treatment and after-treatment cephalometric radiograph tracings. All posttreatment records and Table 1 confirmed improvement of her dental crowding as well as convex profile (ANB = 5°) as well as improvement of her mandibular plan angle (FMA = 35°). Additionally, upper incisor inclination and position relative to SN, NA have been improved close to normal values. In addition, her lower incisor inclination relative to mandibular plane (MP) has been improved (retroclined) to allow the forward position/projection of the mandible. The patient was fitted with four Invisalign Vivera^®^ retainers and was instructed to rotate between them on a monthly basis and to use fulltime for 6 months, in addition to the continuation of using the VPro5 every day as she did during her active treatment period. Then, she was instructed to wear the retainers at night time for the rest of her life as a night guard. 

### 2.2. Case 2

**Figure 2:**
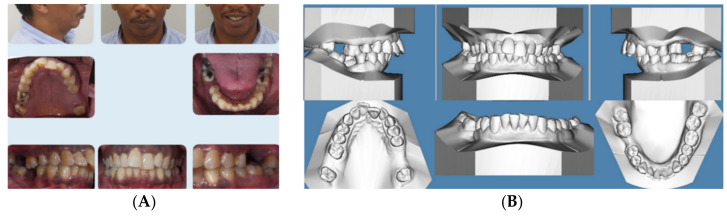

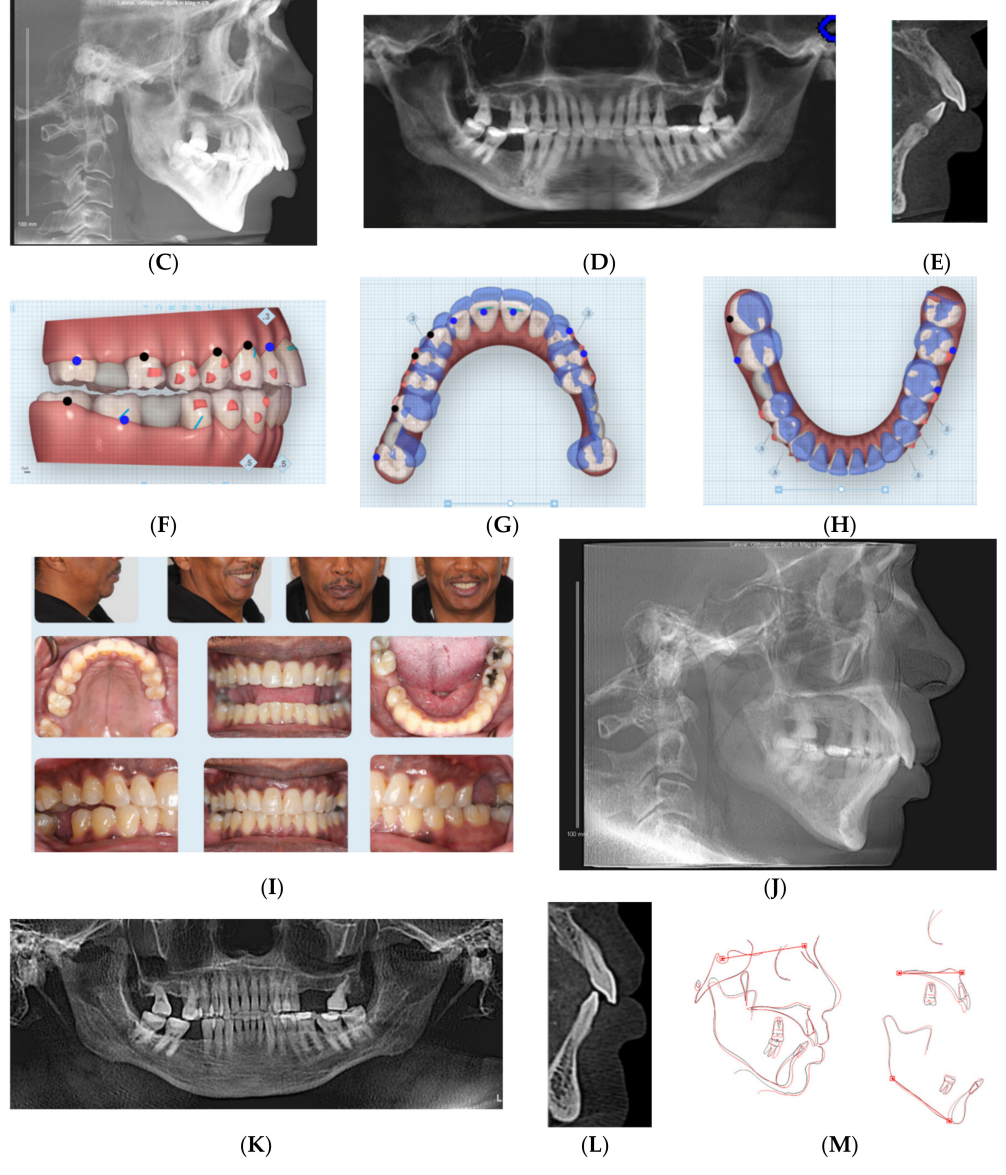
(**A**) Initial photographs showing convex profile with recessive chin and anterior crowding. (**B**) Initial digital models showing left side Class II buccal occlusion with a 7 mm increased overjet. (**C**) CBCT-driven cephalometric radiograph showing convex profile with recessive chin and protrusive upper incisors. (**D**) CBCT panoramic radiograph showing missing 1.7, 2.6, 2.7, and 4.6 molars; no other noticeable intraoral bony lesion could be detected. (**E**) CBCT-driven sagittal screen of lower incisor showing its severe proclination with minimum bone appears on most of the labial surface of the root and compromised crown/root ratios. (**F**) Digital treatment plan (ClinCheck) showing posterior teeth intrusion to help forward rotation of the mandible palatal root torque of upper incisors. (**G**) Upper arch digital treatment plan (ClinCheck) showing expansion (blue—initial teeth position, white—final teeth position) and retraction of upper incisors to improve their initial protrusion. (**H**) Lower arch digital treatment plan (ClinCheck) showing (blue—initial teeth position, white—final teeth position) with interproximal reduction plan to allow lower incisor intrusion and leveling /alignment of lower incisor crowding. (**I**) Final photos showing improved patient’s profile and chin projection as well as aligned upper and lower incisors and Class I buccal occlusion on both sides. Additionally, some remaining posterior open bite due to the posterior teeth intrusion remains; the patient was happy and requested finishing by letting his posterior teeth settle through final retainers. (**J**) CBCT-driven final cephalometric radiograph showing improved profile and chin projection. (**K**) Final CBCT-driven panoramic radiograph. (**L**) Final CBCT-driven sagittal screen radiograph of upper and lower incisors showing improved overjet and bone labial to both upper and lower incisors. (**M**) Superimposition of cephalometric tracing of before (black) and after (red) treatment showing improved forward chin projection, palatal movement of upper incisors, and profile and chin projection and lingual tipping of lower incisors.

This 47-year-old male presented with chief concerns including correcting his crowded teeth (upper arch 5 mm and lower arch 4 mm arch length deficiencies), increased overjet (7 mm), and improving his recessive chin without surgical intervention, if possible. The patient was also not interested in regular fixed appliance. Clinical records (Figure 2A–E) revealed a convex profile with recessive/double chin, skeletal Class II apical base relationship (ANB = 9°) with high mandibular plane angle (FMA = 35°), and compensated upper and lower incisors (U1 NA = 6 mm; U1-SN = 98°; LI NB = 11.3 mm; L1MP −99°). Intraoral photographs and the panoramic radiograph reveal a missing upper right second molar, upper left first and second molars, and lower right first molar. In addition, both panoramic and cephalometric radiographs (Figure 2C,D) reveal a short mandibular ramus and body as well, which makes non-surgical treatment of the case more challenging. Figure 2E shows the crown-root ratio of lower incisors more than 1:1 with thin alveolar bone labio-lingually, which makes it very difficult or risky to move the lower incisors enough to minimize lower arch crowding or minimize overjet should camouflage treatment be proposed.

Treatment plan and progress: The patient was treatment planned using Invisalign clear aligners including upper arch expansion, posterior teeth distalization, as well as intrusion simultaneously to improve overjet and also to allow the forward/vertical bite jump to improve overjet as well as to improve the patient profile. The four digital plans for this patient (ClinCheck^®^) included a total of 140 aligners. Interproximal reduction (IPR) was also prescribed for this patient to minimize black triangles between front teeth as well as to move the upper and lower incisors lingually (Figure 2F–H). Upper incisor lingual movement was planned to minimize the increased overjet, and lower incisor lingual movement and intrusion were performed to minimize anterior interference after the lower jaw was to be rotated forward by posterior teeth intrusion. The patient was not very compliant with daily aligner wear (12–16 h/day) so the VPro5 HFV device was provided to him to use 5 minutes 1-3 times per day. Additionally, the patient was provided with vertical-pull chin cup (Jaw Bra, Chanhassen, MN, USA) to use every night. As a result, he was changing his aligners between 7–10 days based on his comfort with new aligners as well as the new aligners’ maximum fit to his teeth. Light Class II elastics (3/16 3.5 ounces) were provided for him to use during the day and 3/16 4.5 ounces to wear at night) so as not to interfere with his aligner wear. Total treatment time in active aligners was 24 months.

Figure 2I–M and Table 2 show posttreatment records including CBCT-driven cephalometric, panoramic, and sagittal screens of anterior teeth. The patient’s facial convexity has been improved, chief concerns (crowding and increased overjet) have been addressed. Posterior teeth uprighting in preparation of future restoration of missing teeth was achieved. In addition, posterior teeth intrusion to allow forward mandibular rotation was significant to the level it exists on the right side at the final stage. The patient was satisfied, and the slight right posterior open bite was left to settle and to be followed up during the retention phase; however, the patient did not show for follow-up during his retention period.

### 2.3. Case 3

**Figure 3:**
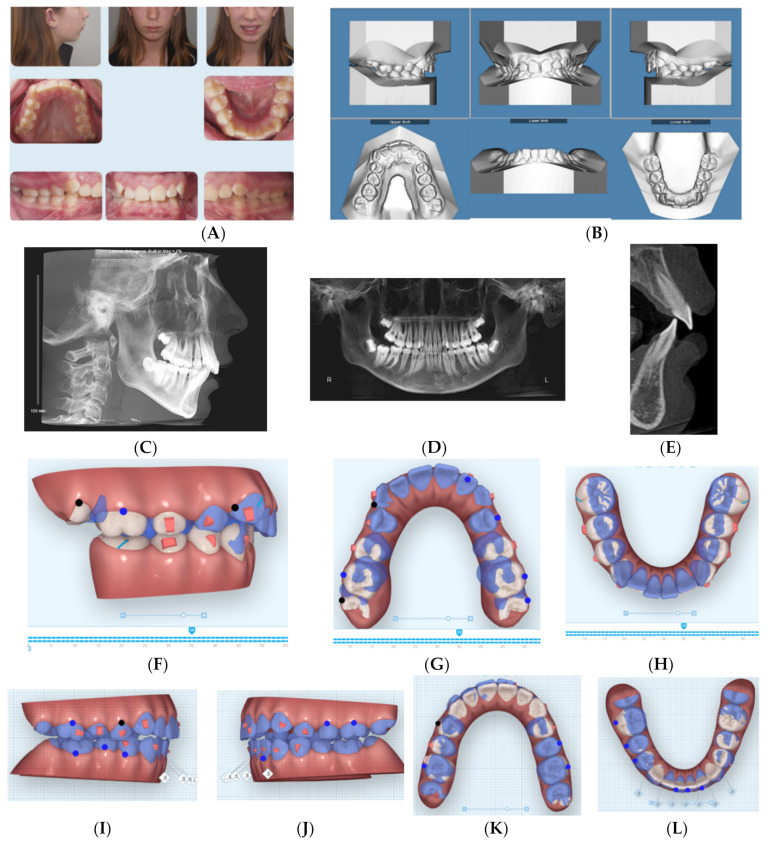

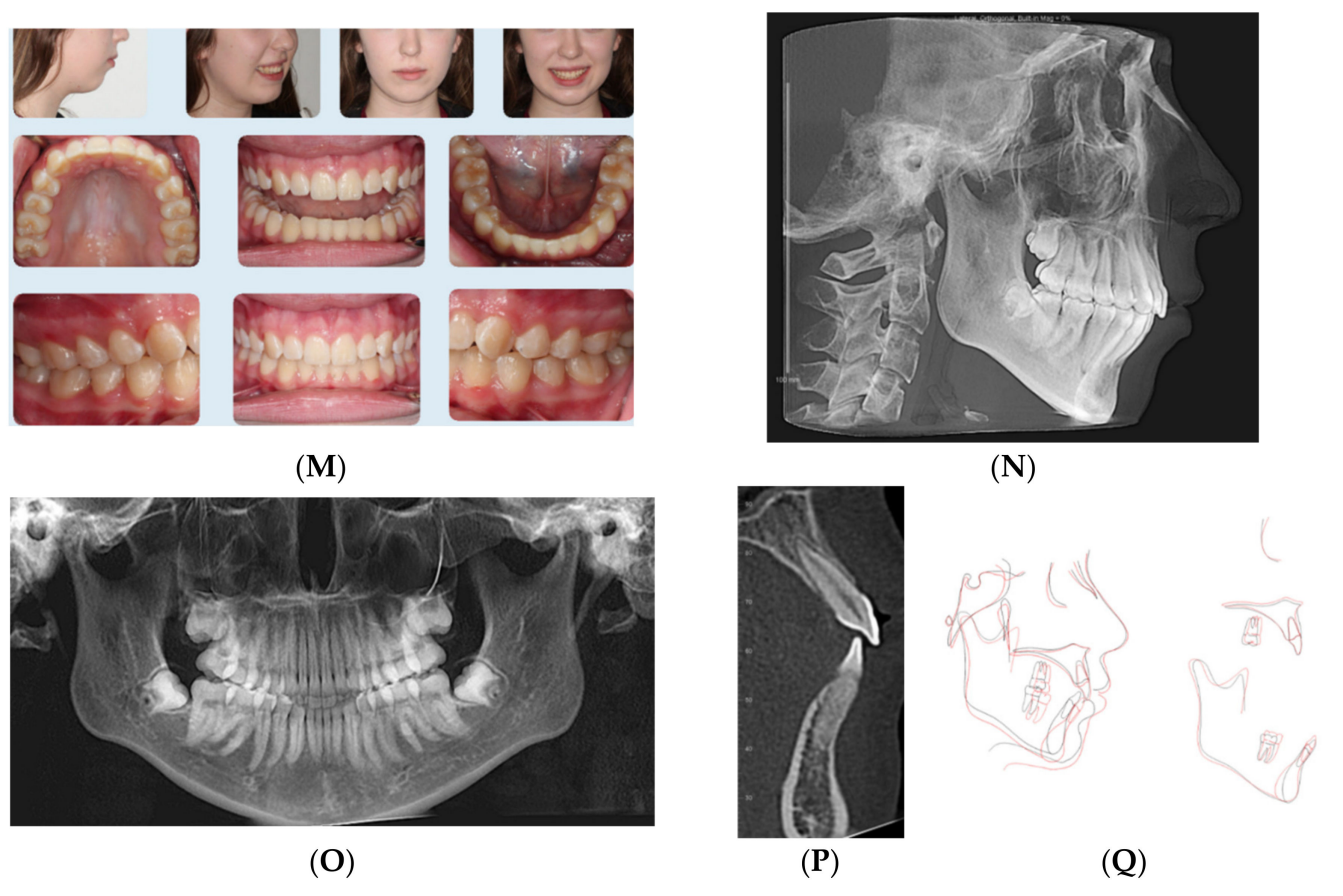
(**A**) Initial photographs showing convex profile with recessive chin and anterior crowding. (**B**) Initial digital models showing Class II Division 2 malocclusion with increased 5 mm overjet and impinging overbite. (**C**) CBCT-driven cephalometric radiograph showing convex profile with recessive chin and protrusive upper incisors. (**D**) CBCT panoramic radiograph showing the presence of permanent teeth; no other noticeable intraoral bony lesion could be detected. (**E**) CBCT-driven sagittal screen of lower incisor showing increased overjet and impinging overbite. (**F**) First digital treatment plan (ClinCheck) showing posterior teeth expansion and distalization. (**G**) Upper arch digital treatment plan (ClinCheck) showing expansion and distalization of posterior teeth (blue—initial teeth position, white—progress teeth position) and retraction of upper incisors to improve their initial protrusion. (**H**) Lower arch digital treatment plan (ClinCheck) showing (blue—initial teeth position, white—progress teeth position) lower arch expansion to allow lower incisor intrusion and leveling/alignment of lower incisor crowding. (**I**,**J**) Second digital treatment plan (ClinCheck) showing posterior teeth intrusion. (**K**) Upper arch digital treatment plan (ClinCheck) showing expansion of posterior teeth (blue—initial teeth position, white—progress teeth position) and retraction of upper incisors to improve their initial protrusion. (K) Lower arch digital treatment plan (ClinCheck) (blue—initial teeth position, white—progress teeth position) showing interproximal reduction between her lower teeth and lower incisor lingual movement to minimize anterior interference during forward rotation of the mandible as planned by the posterior teeth intrusion. (**M**) Final photos showing improved patient’s profile and chin projection as well as aligned upper and lower incisors and Class I buccal occlusion on both sides, improved overbite and overjet. (**N**) CBCT-driven final cephalometric radiograph showing improved profile and chin projection, although not ideal to straight profile, but patient and parents were satisfied with the results compared to initial clinical conditions. (**O**) Final CBCT-driven panoramic radiograph shows growing third molars to be followed for their eruption and possible removal if needed. (**P**) Final CBCT-driven sagittal screen radiograph of upper and lower incisors showing improved overjet and bone labial to both upper and lower incisors. (**Q**) Superimposition of cephalometric tracing of before (black) and after (red) treatment showing improved forward chin projection. However, challenges included vertical displacement of the mandible due to over-eruption of lower molars due to the patient’s mouth breathing habit and non-compliance of using the vertical-pull chin cup/Jaw Bra. Additionally, it shows lingual tipping of lower incisors.

This 14-year-old female presented with chief concerns including severe dental crowding, increased overbite and overjet, and recessive chin. She and her parents were not interested in any surgical intervention for her recessive chin. Her medical history was not contributory, and her growth spurt was already passed as confirmed from her maturity indicator from CVM and physical/menstrual history. Her parents reported that she is a mouth breather and opens her mouth during sleep. Clinical records (Figure 3A–E) and cephalometric analysis (Table 3) revealed a convex profile with recessive chin. 

The patient has a dolichofacial type with compensated Class II skeletal base relationship (ANB = 8.4°, SNB = 74.1°; FMA = 36.4°). Intraoral photos and digital models revealed a Class II division 2 malocclusion with increased overjet (5.5 mm) and impinging overbite (95%), as well as moderate upper and lower dental crowding (5 mm in each arch). 

Treatment plan and progress: The patient and parents were made aware of the challenges of mouth breathing habit and its detrimental effects on treatment outcomes and stability. In addition, myofunctional therapy was proposed using a trainer for braces (T4B, Myobrace, Rancho Cucamonga, CA, USA) that was prescribed to be worn one hour during the day and night time with vertical-pull chin cup (Jaw Bra, Chanhassen, MN, USA). The treatment plan included sequential distalization of the upper posterior teeth utilizing light (3/16 3.5 ounce) Class II elastics for inter-maxillary anchorage and torqueing of upper incisors’ roots palatally. The vertical-pull chin cup together with occlusal coverage of the posterior teeth with the Invisalign aligners (working as posterior bite blocks) were intended to intrude posterior teeth. The first batch of aligners included 69 aligners; however, at stage 35 (Figure 3F–H), the patient was supposed to have molars in an end-to-end relationship, but due to the patient’s non-compliance, a decision was made to rescan the patient’s teeth for additional aligners that revealed the Class I molars over correction (Figure 3I–L). At that time, the patient was provided with VPro5 to use for 5 min per day to help with aligner seating if the patient experienced pain or if the aligners became ill-fitting due to long periods of time she was not wearing aligners during the day (12–16 h per day aligner wear per her report). The patient also could not use the Jaw Bra as prescribed, so the new additional aligners incorporated sequential posterior teeth intrusion to allow forward rotation of the mandible. In addition, the upper inter-premolars distances were expanded to correct the crossbite observed at stage 35 of the first batch of aligners (35/69). Lower incisor retroclination was prescribed using IPR of the lower incisors to help with minimizing anterior interference when the mandible was to be auto-rotated forward. When the patient used VPro5, she was able to change her aligners every 5–7 days considering her noncompliance with daily aligner wear (12–16 h of daily aligner wear). 

When she used VPro5, her total treatment time was one year. Final records (Figure 3M–Q) showed maintenance of her vertical dimension (no change of FMA (Table 3)), improved apical-base relationship (ANB = 6.7°), lower incisor retroclination (IMPA = 87.5°). Although her mandibular apical base was moved forward (SNB = 76.7°), this improvement was counteracted by the lower posterior teeth extrusion (Figure 3Q) due to noncompliance with the Jaw Bra and existence of the mouth-breathing habit especially at night time. Overall superimposition shows the downward displacement of the mandible, which was due to extrusion of lower molars that could have been prevented by using the Jaw Bra.

### 2.4. Case 4

This 14-year-old female presented with chief concerns that she wanted to improve her crowded teeth and deep bite as well as improve her recessive chin as much as possible without surgical intervention. The patient maturity indicator from CVM and physical/menstrual history confirmed that she already passed her growth spurt.

**Figure 4:**
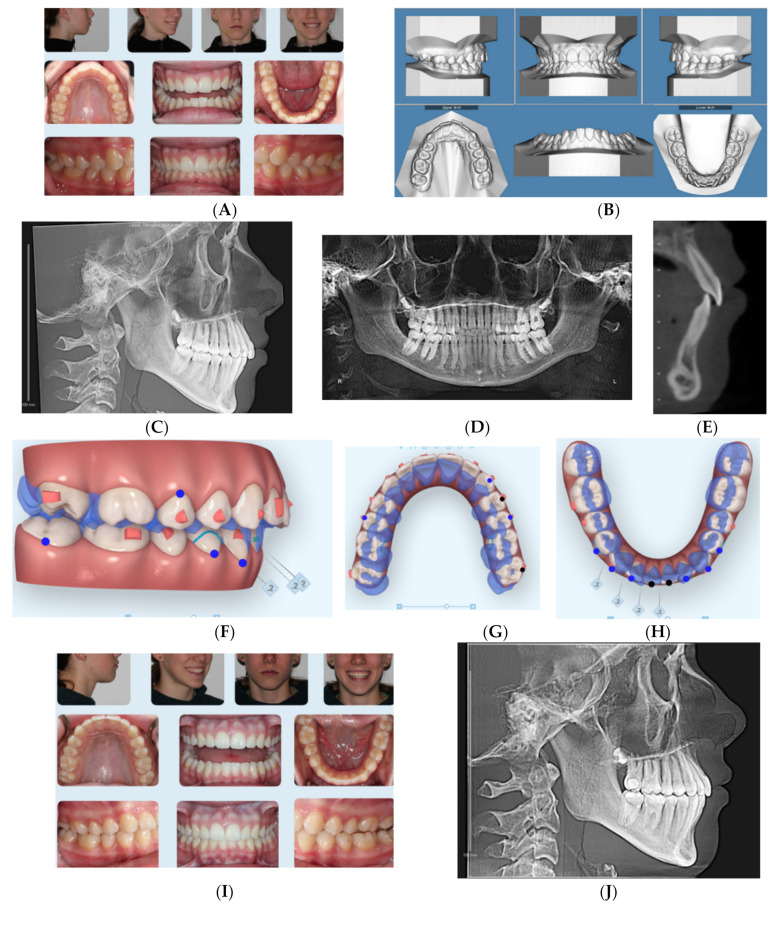

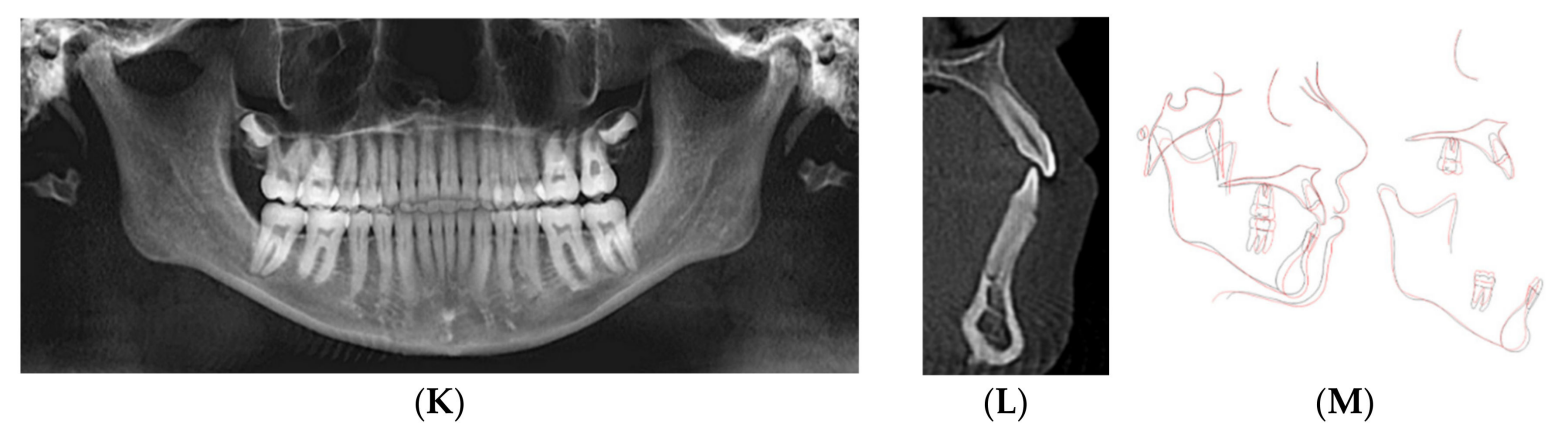
(**A**) Initial photographs showing convex profile with recessive chin and anterior crowding. (**B**) Initial digital models showing Class II Division 2 subdivision left side malocclusion with increased overbite and excessive curve of Spee. (**C**) CBCT-driven cephalometric radiograph showing convex profile with recessive chin and normally positioned upper incisors/nasolabial angle. (**D**) CBCT panoramic radiograph showing presence of all permanent teeth except lower third molar germs; no other noticeable intraoral bony lesion could be detected. (**E**) CBCT-driven sagittal screen of lower incisor showing increased overbite. (**F**) The digital treatment plan (ClinCheck) showing posterior teeth intrusion to help forward rotation of the mandible. (**G**) Upper arch digital treatment plan (ClinCheck) showing expansion and labial movement of the upper arch (blue—initial teeth position, white—final teeth position). (**H**) Lower arch digital treatment plan (ClinCheck) showing expansion (blue—initial teeth position, white — final teeth position) and lower anterior interproximal reduction to allow lower incisor intrusion and leveling /alignment of lower incisors. No attempt was made to procline the lower incisors. (**I**) Final photos showing improved patient’s profile and chin projection as well as aligned upper and lower incisors and Class I buccal occlusion both sides. Additionally, over bite has been improved compared to initial records. (**J**) CBCT-driven final cephalometric radiograph showing improved profile and chin projection. (**K**) Final CBCT-driven panoramic radiograph. (**L**) Final CBCT-driven sagittal screen radiograph of upper and lower incisors showing improved overbite and bone labial to both upper and lower incisors. (**M**) Superimposition of cephalometric tracing of before (black) and after (red) treatment showing improved forward chin projection, palatal movement of upper incisors roots while upper molars and incisors crowns moved forward by the dental decompensation, and profile and chin projection and lingual tipping of lower incisors.

The patient was not interested in a fixed orthodontic appliance. Clinical records (Figure 4A–E) and Table 4 show the convex profile with recessive chin (ANB = 4.2°, SNB = 78.5°), left side occlusion was end-to -end and the right side was Class I with deep overbite (95%), with excessive curve of Spee and 3 mm overjet. She had no contributory medical or dental history to her current malocclusion.

Treatment plan and progress: The minimum overjet was challenging to allow the mandible to be projected forward, so dental decompensation protocol was applied to this patient. In other words, treatment planning included advancing the upper arch forward using class III elastics (3/16-4.5 ounces full time) as well as expanding the upper arch at the same time in addition to intruding posterior teeth as moving the upper arch forward. Another challenge in this case was the excessive curve of Spee with moderate lower incisor crowding (6 mm). So in order to advance the lower arch, the excessive curve of Spee should be leveled, also due to the high angle presentation of this case (FMA = 30.4° Table 4). Another challenge in the lower arch was the lower incisor inclination (L1 MP = 84.9o). Hence, the initial goal was to move lower incisors slightly lingually to minimize anterior interference once the mandible is rotated forward or at least maintaining their original position. In addition, the retroclined upper incisors (U1-SN = 100.6o) would allow forward positioning/proclination of upper incisors. Treatment planning also included utilization of Class III elastic button cutouts to the upper first molars palatally to assist with upper arch expansion as well as lower first premolars (3/16-4.5 ounces) full time. She was provided with a VPro5 appliance and directed to use it for 5 minutes every day. Additionally, the patient was provided with vertical-pull chin cup (Jaw Bra, Chanhassen, MN USA) to use every night. She was able to change her aligners every 4–5 days.

Her treatment involved 23 aligners, and no additional aligners were needed afterwards. Treatment time was 4 months. Then she was given 4 sets of Vivera retainers. Final records were taken at one year from the original records that show better chin-forward projection (Figure 4I–M) (Table 4). Superimposition of before and final CBCT-driven cephalometric radiographs show the forward projection of the chin and the decompensated upper and lower incisors.

## 3. Results

General results in all cases included improved facial convexity (Table 1, Table 2, Table 3 and Table 4) and (Figure 1N, Figure 2M, Figure 3Q, and Figure 4M) by forward projection of the chin due to auto-rotation of the mandible produced by posterior teeth intrusion. The use of vertical-pull chin cup (Jaw Bra) helped with posterior intrusion except for the patient 3 that was not very compliant in wearing either the aligners or the Jaw Bra. Regardless, her results are acceptable, although she did not end up having a straight profile. It is important to elaborate on the lower incisor intrusion and lingual movement to facilitate the forward rotation of the mandible by eliminating anterior interference. In cases where lower anterior teeth are crowded, flared, or extruded as in all the presented cases here, gaining spaces to solve these issues could be achieved by lower arch expansion and/or interproximal reduction of lower teeth. It is to be noted that if lower arch expansion is to be performed, final upper and lower arches should be coordinated, especially after forward rotation of the mandible. Retention is considered wearing the vivera retainers full time for a year and night time forever.

## 4. Discussion

The increased demand of non-surgical techniques to correct skeletal mal-relationship cases especially in non-growing or slow growing patients is increasing [25,26]. In this study, it has been shown that the use of HFV enhances treatment outcomes in challenging high-angle orthodontic cases when normally, surgical options would have been suggested to manage these cases. The results of this study showed improvement of these non-growing patients’ profiles non-surgically. HFV has been chosen for these patients because of its ease-of-use and patient acceptance. Photobiomodulation as well as low-frequency vibration and low intensity pulsed ultrasound require longer daily treatment times of 10-20 minutes [7,8,10,11]. Additionally, HFV has been previously shown to accelerate orthodontic treatment especially with clear aligners due to the proposed hypothesis that HFV helps seat clear aligners and encourages high patient compliance [15,16,17]. In addition, it helps with osteoblastic/osteoclastic activities within the alveolar bone as well as increased bone density during passive force or in the absence of static force [17,18,19,20]. It is of particular importance to note that adult or non-growing patients would need relatively longer treatment time than growing individuals [27]. In particular, camouflage treatment or non-surgical treatment in skeletal mal-relationship cases [25] would be needed. The relatively faster treatment in the presented cases in this report could be due to the reported scientific evidence that HFV enhances bone resorption during orthodontic treatment as well as bone deposition. This agrees with previous reports [15,16,17,18,19,20,21,22,23]. In addition, the appearance of bone labial to lower incisors after moving them lingually in almost all cases supports the stimulatory effect of HFV on bone formation. This is in agreement with a pervious case report [28]. Due to the relatively wide range of patients’ ages presented in this study, results should be interpreted with care; however, the very short treatment time in younger patients (Cases 3, 4) compared to cases 1 and 2 is also in agreement with the previous report that regular orthodontic treatment time is longer with the patient’s increasing age. Future research should be planned in such way to match the difficulty of cases, age, and gender between the HFV and control (no HFV) cases. The concept of decompensating upper and lower incisors to allow forward rotation of the mandible by posterior teeth intrusion is supported in this study using HFV as seen on posttreatment sagittal CBCT screens. It is to be noted that lower incisors forward inclination can lead to long-term gingival recession [29]. The application of HFV together with moving lower incisors lingually in compensated skeletal Class II cases may help to prevent gingival recession because of the documented ability of HFV to increase bone formation [10,11,12,13,14,15,16,17,18]. The use of jaw bra type of vertical pull chin cup to control the vertical dimension is new to report her. Similar vertical control has been reported previously during distalization using clear aligners [30]. One limitation of this study is that there were no control cases to demonstrate what could have occurred if the VPro5 device was not used. Another limitation is the small sample size presented in this study; hence, more details to statistically determine an appropriate sample size should be planned in the future. These limitations can be addressed in prospective randomized controlled clinical trials in the future. However, as a case presentation, this study has clinical values to help clinicians to have more options when they are presenting patients with different plans of treatment.

## 5. Conclusions

1. The use of HFV concurrent with SmartTrack^®^ Invisalign aligners and vertical-pull chin cup in this case series allowed achievement of complex tooth movement including posterior teeth intrusion and expansion simultaneously as well as forward mandibular rotation/projection in non-growing patients with a skeletal Class II relationship, which otherwise would have required surgery. These results should be confirmed with future larger samples size prospective controlled clinical trials.
2. Although the improvement of patients’ profiles was not as ideal as could possibly achieved with surgical intervention, the achieved improvement was satisfactory to all patients/parents.
3. The clinical impact and implications of this case series are that the use of HFV facilitates complex orthodontic tooth movement including posterior teeth intrusion and incisor decompensation in addition to increasing bone formation labial to lower incisors that may minimize future gum recession due to their labial inclination. Again, the presented results should be confirmed with future larger samples size prospective controlled clinical trials.

## Figures and Tables

**Table 1:** Before-and-after treatment cephalometric analyses of case 1.

Measurement	Initial	Final	Change/Variation from Initial Values	Normal
SNA (º)	87.1	87.5	0.4	82
SNB (º)	79.3	82.5	3.2	80.9
SN-MP (º)	41.8	40	−1.8	32.9
FMA (MP-FH) (º)	38.1	35.5	−2.6	23.9
ANB (º)	7.9	5	−2.9	1.6
U1-NA (mm)	0.5	1.3	0.8	4.3
U1-SN (º)	92	99	7	102.8
L1-NB (mm)	8.1	4.8	−3.3	4
L1-MP (º)	90.4	88.6	−1.8	95
Lower Lip to E-Plane (mm)	4.9	2.2	−2.7	−2
Upper Lip to E-Plane (mm)	4.3	0.7	−3.6	−6
Nasolabial Angle (Col-Sn-UL) (º)	107.4	109	1.6	102
Facial Convexity (G’-Sn-Po’) (º)	29.5	22.7	−6.8	12

**Table 2:** Before-and-after treatment cephalometric analyses of Case 2.

Measurement	Initial	Final	Change/Variation from Initial Values	Norm
SNA (º)	83.7	83.6	−0.1	82
SNB (º)	74.7	76.3	2.4	80.9
SN-MP (º)	41.3	41	0.3	32.9
FMA (MP-FH) (º)	35.2	37.1	1.9	22.9
ANB (º)	9	7.3	−1.7	1.6
U1-NA (mm)	5.9	3.5	−2.4	4.3
U1-SN (º)	98.4	90.4	2	103.1
L1-NB (mm)	11.3	9.4	−1.9	4
L1-MP (º)	99.2	88.2	−11	95
Lower Lip to E-Plane (mm)	9.7	6.4	−3.4	-2
Upper Lip to E-Plane (mm)	7.1	3.2	−3.9	-8
Nasolabial Angle (Col-Sn-UL) (º)	104.3	107.2	2.9	102
Facial Convexity (G’-Sn-Po’) (º)	22.7	20.6	−1.9	12

**Table 3:** Before-and-after treatment cephalometric analyses of Case 3.

Measurement	Initial	Final	Change/Variation from Initial Values	Norm
SNA (º)	82.5	83.3	0.8	82
SNB (º)	74.1	76.7	1.4	80.9
SN-GoGn (º)	41.1	41.1	0	32.9
FMA (MP-FH) (º)	36.4	36.3	−0.1	24.9
ANB (º)	8.4	6.7	−1.4	1.6
U1-NA (mm)	−1.8	0.7	2.5	4.3
U1-SN (º)	89.1	92.4	3.3	102.5
L1-NB (mm)	6.7	8.1	1.4	4
L1-GoGn (º)	95.2	87.5	−7.7	93
Lower Lip to E-Plane (mm)	0.7	3.6	2.9	-2
Upper Lip to E-Plane (mm)	0.8	0.8	0	-3.9
Nasolabial Angle (Col-Sn-UL) (º)	105	105.6	0.6	102
Facial Convexity (G’-Sn-Po’) (º)	23.4	21.6	−1.8	12

**Table 4:** Before-and-after treatment cephalometric analyses of Case 4.

Measurement	Initial	Final	Change/Variation from Initial Values	Norm
SNA (º)	82.9	81.4	−1.5	82
SNB (º)	78.5	79	0.5	80.9
SN-MP (º)	37.5	37.7	0.2	32.9
FMA (MP-FH) (º)	30.4	28.7	−2.3	24
ANB (º)	4.5	2.4	−2.1	1.6
U1-NA (mm)	4.2	5.7	1.5	4.3
U1-SN (º)	100.6	102.2	1.6	102.8
L1-NB (mm)	7.4	5.8	−1.6	4
L1-MP (º)	84.9	81.8	−3.1	95
Lower Lip to E-Plane (mm)	1.4	1	−0.4	−2
Upper Lip to E-Plane (mm)	−2.7	−0.9	−1.6	−5.7
Nasolabial Angle (Col-Sn-UL) (º)	111.2	107.2	−3	102
Facial Convexity (G’-Sn-Po’) (º)	21.2	16.5	−4.7	12
